# Supplementary material for: The BBS/CCT chaperonin complex ensures the localization of the adhesion G protein-coupled receptor ADGRV1 to the base of primary cilia
Source: Front Cell Dev Biol. 2025 Mar 4;13:1520723. doi: 10.3389/fcell.2025.1520723 (PMC11913874; doi:10.3389/fcell.2025.1520723)
Supplement: Supplementary file 3 [file DataSheet1.pdf]

The BBS/CCT chaperonin complex ensures the localization of the adhesion G protein-coupled receptor ADGRV1 to primary cilia

Joshua Linnert[1*], Deva Krupakar Kusuluri[1*], Baran E. Güler[1], Sarita Rani Patnaik[2], Helen Louise May-Simera[2], Uwe Wolfrum[1,3$]

[1]Institute of Molecular Physiology, Molecular Cell Biology, Johannes Gutenberg University Mainz, Germany. [2]Institute of Molecular Physiology, Cilia Biology, Johannes Gutenberg University Mainz, Germany; [3]Institute for Quantitative and Computational Biosciences (IQCB), Johannes Gutenberg University Mainz, Germany.

*Authors contributed equally to this work

$Corresponding author: Institute of Molecular Physiology, Molecular Cell Biology, Johannes Gutenberg University Mainz, Hanns-Dieter-Hüsch-Weg 17, 55128 Mainz, Germany; e-mail: wolfrum@uni-mainz.de

Supplementary Materials:

Supplementary Table S1: Compiled ciliary proteome

Supplementary Table S2: Ciliary proteins in TAP data sets

Supplementary Table S3: Gene Ontology (GO) term analysis of DEGs in USH2C patient-derived fibroblasts

Supplementary Table S4: Gene Ontology (GO) term analysis of DEGs in Adgrv1/delTM mouse retinae

Supplementary Figure S1: Validation of siRNA-mediated knockdown chaperonins *BBS6*, *CCT2*, and *CCT3*.

Supplementary Figure S2: Unaltered ciliary localization of CCT3 after siRNA-mediated *BBS6* knockdown.

Supplementary Figure S3: Supplementary Figure S3. Primary cilia length is in CCT2 and CCT3 depleted hTERT-RPE1 cells.

Supplementary Figures

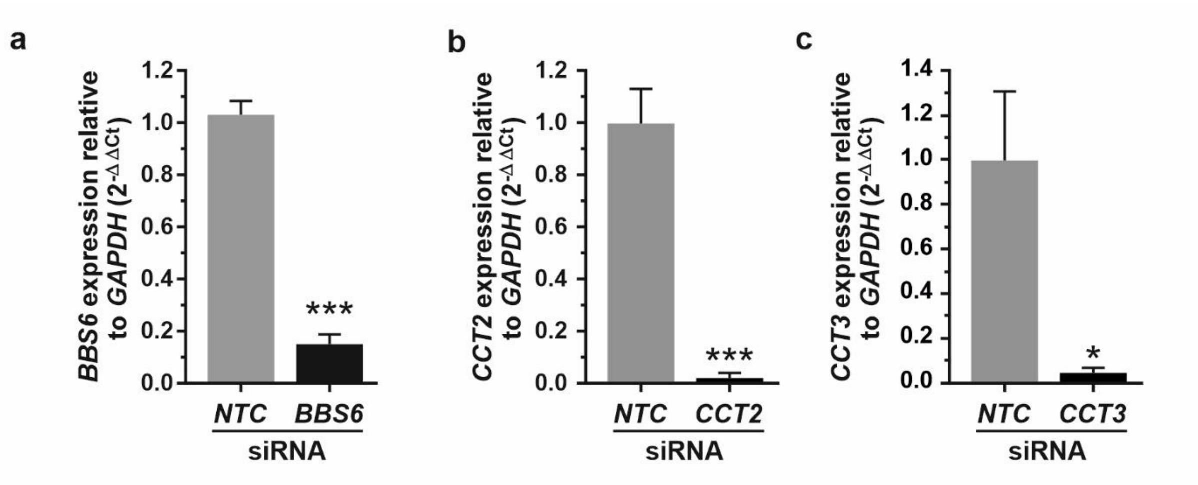

Supplementary Figure S1. Validation of siRNA-mediated knockdown chaperonins *BBS6*, *CCT2*, and *CCT3*.

(**a-c**) Analysis of mRNA expression levels after siRNA-mediated knockdown (KD) of *BBS6* (**a**), *CCT2* (**b**), and *CCT3* (**c**) compared non-targeting (NTC) siRNA controls in hTERT-RPE1 cells by quantitative real-time PCR (qRT-PCR). The expression of all three mRNAs are significantly downregulated. Statistical significance was determined by the two-tailed Student's *t*-test (a-c): *p < 0.05, **p < 0.01, ***p < 0.005.

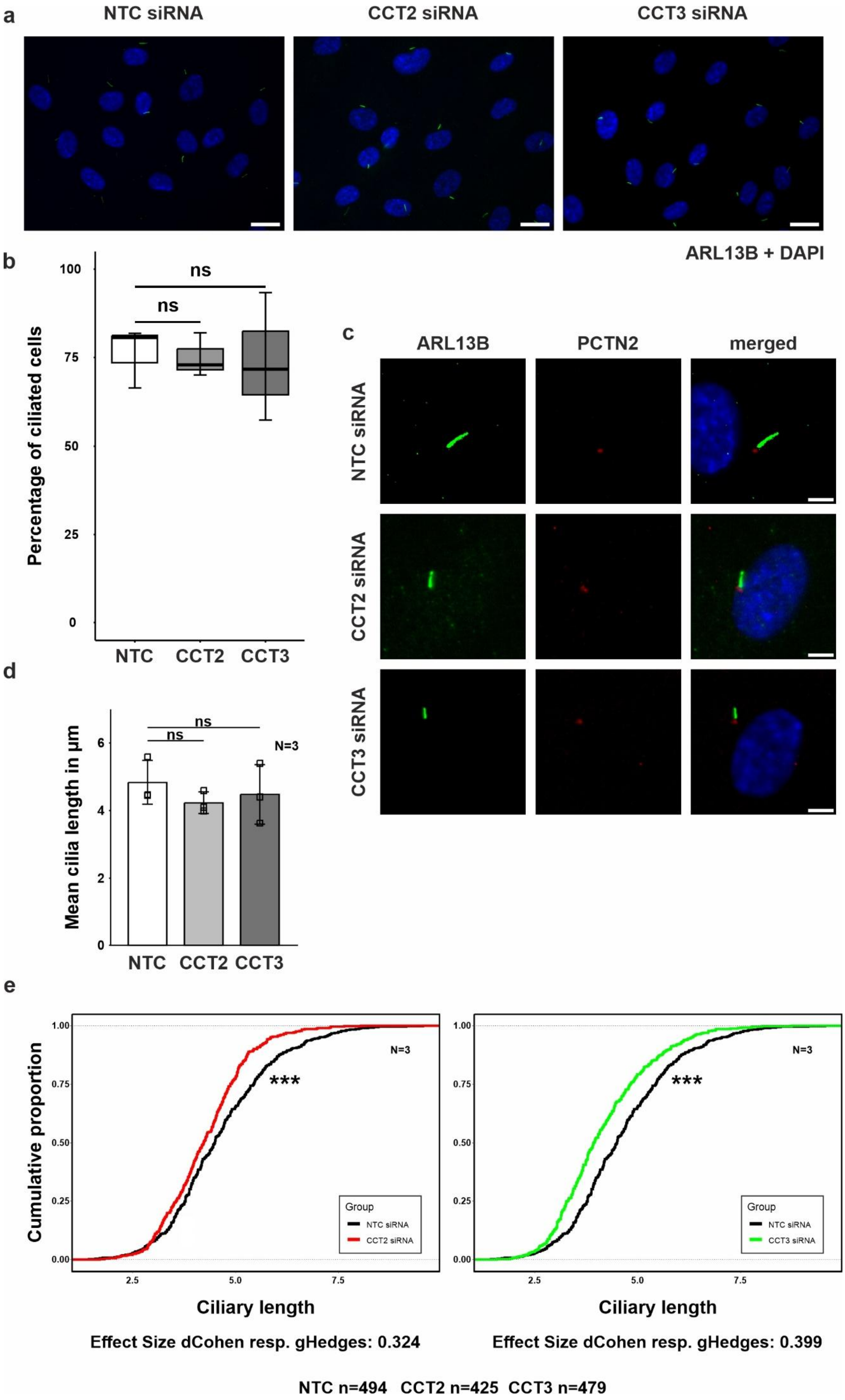
a
NTC siRNA
CCT2 siRNA
CCT3 siRNA
ARL13B + DAPI
b
Percentage of ciliated cells
100
75
50
25
0
ns
ns
NTC
CCT2
CCT3
c
ARL13B
PCTN2
merged
NTC siRNA
CCT2 siRNA
CCT3 siRNA
d
Mean cilia length in µm
6
4
2
0
ns
ns
N=3
NTC
CCT2
CCT3
e
Cumulative proportion
1.00
0.75
0.50
0.25
0.00
N=3
***
Group
NTC siRNA
CCT2 siRNA
CCT3 siRNA
2.5
5.0
7.5
Ciliary length
Effect Size dCohen resp. gHedges: 0.324
Effect Size dCohen resp. gHedges: 0.399
NTC n=494 CCT2 n=425 CCT3 n=479

Supplementary Figure S2. Primary cilia length is in CCT2 and CCT3 depleted hTERT-RPE1 cells.

(**a**) Low magnification of immunofluorescence images of hTERT-RPE1 cells depleted for CCT2 and CCT3 by siRNA-mediated knockdown stained for the cilia marker ARL13B. (**b**) Quantification of ciliated hTERT-RPE1 cells (**c**) Immunofluorescence labelling of the cilia marker ARL13B and the ciliary base marker pericentrin 2 (PCTN2) in hTERT-RPE1 cells which were depleted for CCT2 and CCT3 by siRNA-mediated knockdown. (**d**) Quantification of mean cilia length showed no significant differences compared to NTC control. (**e**) Evaluation of the distribution of the cilia length however showed a significant reduction of long cilia in CCT2 and CCT3 depleted cells. N = number of biological replicates, n = number of analysed cells. Statistical significance was determined by the two-tailed Student's *t*-test (b,d) and the Kolmogorov-Smirnov test (e): $*p < 0.05$, $**p < 0.01$, $***p < 0.005$. Scale bar: a = 20 µm, c = 5 µm.

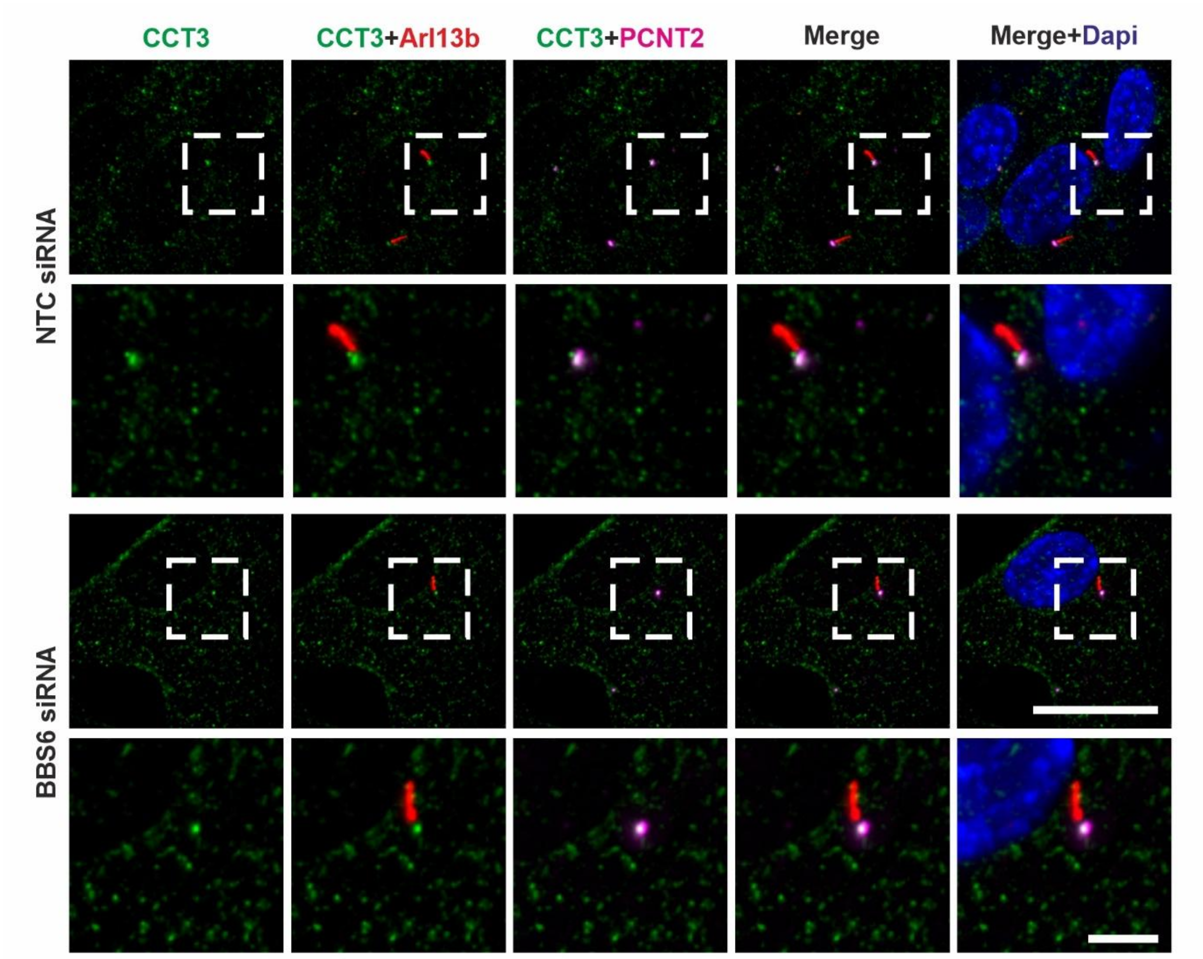

Supplementary Figure S3. Unaltered ciliary localization of CCT3 after siRNA-mediated *BBS6* knockdown.

Representative immunofluorescence images of hTERT-RPE1 cells labelled with antibodies against ARL13B as ciliary shaft marker (red), (PCNT2; magenta) as ciliary base marker, and CCT3 (green) to the base of the cilium after siRNA-mediated *BBS6* knockdown (KD) in comparison to control cells. Nuclear DNA is counterstained by DAPI (blue). The ciliary base localization of CCT3 is not altered in *BBS6* KD cells. Scale bar: 25 µm and 5 µm in magnified images.
